# Observational and Accelerometer Analysis of Head Movement Patterns in Psychotherapeutic Dialogue ^†^

**DOI:** 10.3390/s21093162

**Published:** 2021-05-02

**Authors:** Masashi Inoue, Toshio Irino, Nobuhiro Furuyama, Ryoko Hanada

**Affiliations:** 1 Department of Information and Communication Engineering, Tohoku Institute of Technology, 35-1 Yagiyama Kasumicho, Taihaku-ku, Sendai 982-8577, Japan; 2 Faculty of Systems Engineering, Wakayama University, Sakaedani 930, Wakayama City 640-8510, Japan; irino@wakayama-u.ac.jp; 3 Faculty of Human Sciences, Waseda University, 2-579-15 Mikajima, Saitama, Tokorozawa City 359-1192, Japan; furuyama@waseda.jp; 4 Department of Psychology, Tokyo Woman’s Christian University, 2-6-1 Zempukuji, Suginami-ku, Tokyo 167-8585, Japan; hanada@lab.twcu.ac.jp

**Keywords:** head nod, head movement, dialogue progression, psychotherapy, face-to-face counseling, case study

## Abstract

Psychotherapists, who use their communicative skills to assist people, review their dialogue practices and improve their skills from their experiences. However, technology has not been fully exploited for this purpose. In this study, we analyze the use of head movements during actual psychotherapeutic dialogues between two participants—therapist and client—using video recordings and head-mounted accelerometers. Accelerometers have been utilized in the mental health domain but not for analyzing mental health related communications. We examined the relationship between the state of the interaction and temporally varying head nod and movement patterns in psychological counseling sessions. Head nods were manually annotated and the head movements were measured using accelerometers. Head nod counts were analyzed based on annotations taken from video data. We conducted cross-correlation analysis of the head movements of the two participants using the accelerometer data. The results of two case studies suggest that upward and downward head nod count patterns may reflect stage transitions in counseling dialogues and that peaks of head movement synchrony may be related to emphasis in the interaction.

## 1. Introduction

It is essential for psychotherapists to review their dialogue practices and improve their communicative skills to assist people in need. For the purpose of reflection, hand-written notes and voice recorders have typically been used as memory aids; for example, voice recordings were used by Rogers [1]. Video-based reflection has also been used in psychotherapy [2]. By reviewing videos, practitioners can discover unnoticed behavior qualitatively. However, the procedure for quantitative analysis of non-verbal behavior during an entire interview session is still being explored. Although non-verbal behavior has been emphasized to be important for establishing therapist–client relationships, this is taught conceptually (i.e., in terms of attitudes rather than specific actions). Therefore, many practitioners—even experienced ones—may find it difficult to observe their own or their colleagues’ dialogue videos analytically from a non-verbal point of view. In this study, we describe a method of analyzing face-to-face interactions to identify structural patterns in dialogues.

Our focus is on head gestures, especially nodding. We quantitatively analyze the use of head nods in dialogues. Nodding is important because it is strongly associated with empathy, which is the basis of bonding; among the various head movements, it has received considerable attention [3]. In psychotherapy, head nodding is considered to be a non-verbal element that is particularly related to the clients’ reaction [4]; in fact, it is observed that a head nod increases the clients’ references to their feelings [5]. Its role is often taught during therapist training. However, few studies have conducted qualitative analysis on the time course, the varying behavior over time, of gestures such as head nodding. As Rogers indicated [6], a therapist must listen to the client skillfully for the counseling to be successful. We expect that the time course of the nodding pattern would give an impression that the client is being assisted by the therapist. We measure head nodding counts and their timings corresponding to the changes in state of the dialogue. Manual annotations by a non-participating psychotherapist and a mechanical recording of head movements using an accelerometer are used to analyze the relationship between the participating therapists and the clients. It is argued that it is important to collect data on head movement directly without human interpretation [7]. Therefore, in addition to the manual annotation of head nods, accelerometers are introduced for objective data acquisition.

Accelerometers have been used in various applications including the head motion-based input device for video games [8], analysis of tennis playing (when mounted on the arm) [9], fall detection of elderly people (when mounted on the waist) [10] or on the head [11]) and tracking human activities within the home environment [12]. An accelerometer can be used with other sensors as an inertial-measurement-unit (IMU) [13]. IMUs can be further combined to track human motions such as with ranging sensors [14] or Global Navigation Satellite System [15]. In human communication analysis, sensors other than accelerometers were used. For example, Kinect sensors have been used to analyze student presentation [16]. In the healthcare domain, accelerometers have been used to detect heart infarction [17] and health issues in pigs [18]. Further, they have been used to measure some aspect of physical activities such as intensity [19,20]. We use them to measure the behavior of interlocutors in the mental health domain and compare the information obtained from accelerometers with those of manual annotation from videos.

In the field of psychology, head nodding in dialogues has been studied as a non-verbal behavior; it has been found to extend the duration of speech [21] and contribute to taking turns [22]. These studies were either experimental or involved summarizations of entire dialogues. Counseling often has a more complex structure than medical diagnostics because of the higher level of interaction involved; for example, clients give feedback to therapists’ interpretation of their problems [23]. Therefore, we should observe the entire dialogue to understand the complex interaction during psychotherapeutic interviews. Studies on head nodding for goal-oriented dialogues exist, such as those between hairdressers and customers; however, they are mostly qualitative [24]. We study the time course of nodding patterns quantitatively during actual task-oriented dialogues using detailed manual annotations and objective sensor data. In this paper, we describe our method of analyzing psychotherapeutic dialogue and the results of the case studies we conducted to analyze such dialogue. Our approach is different from the existing methods in that the accelerometers were used specifically to measure head movements during the dialogue session and the utility of the sensor-based method were compared with manually annotated head nod information. Our approach has an advantage over video processing-based techniques in that accelerometers can obtain head movement information directly. Although there are studies combining vision-based methods with sensor-based methods for improving activity recognition [25], we examined observational analysis on videos and signal processing using accelerometers separately, considering their subjective and objective properties.

The difficulty in analyzing actual, not simulated, psychotherapeutic dialogue is that the interview is conducted only once, and the same dialogue is not repeated. Therefore, two approaches are generally followed. The first is to maintain the real and natural aspect of the dialogue and analyze each unique case. The other is to extract the essence of psychotherapeutic sessions and establish an experimental setting by controlling topics and the backgrounds of the speakers, to record the dialogue repeatedly under the same conditions. In this paper, we took the first approach and describe the method and results of two real interview sessions. This paper is organized as follows. Section 2 explains the materials and methods used. Then, Section 3 presents the results of the case analysis. Section 4 discusses the automation of annotation, different ways of reflections in psychotherapy, and the feedback to practitioners. Finally, Section 5 concludes the paper.

## 2. Materials and Methods

### 2.1. Data Collection

We recorded and analyzed two counseling interviews with different therapists, clients, and client problems. The dialogues were recorded at Kyoto University of Education. The interviews were recorded on different dates and completed in a single session. These interviews were not set up for the purpose of our experiment; the participants were students who created a learning group and conducted training counseling interviews themselves. Typical counseling usually requires multiple interviews; however, the sessions in this learning group were completed in single interviews for ease of organization. The length of the interview was not pre-determined and could be controlled by the participants. In the interviews, actual problems were discussed; the interviews were not role-plays using specific roles and scenarios. All participants were native Japanese speakers and communicated in Japanese. The data used in this study were made available to us by the participants via signed consent forms for research purposes.

The properties of the datasets are listed in Table 1. Throughout both sessions, the participants worked on their problems seriously and eagerly. The first counseling session was carried out by a novice therapist, who was a graduate student studying psychotherapy. This therapist had just been taught a course on the general concepts of psychotherapy and was not based at any psychotherapy school. The second session was conducted by an expert therapist who supervised students majoring in psychotherapy. This therapist was specialized in family/brief therapy that focuses on quick problem solving. Therefore, the background of the therapist may have influenced the dialogue strategy and length of the session. For convenience, in our research, therapists with more than three years of experience after qualification were referred to as *experienced*; therapists with less experience were referred to as *novice*. Based on this definition, the first session therapist was considered to be novice and the second session therapist was considered to be experienced, as shown in Table 1. Both sessions were completed successfully but in different ways. In the first session, the client felt that he could organize their ideas and felt relieved after talking. In the second counseling session, the therapist proposed an intervention, and the client readily agreed.

#### Data Acquisition

**Figure 1:**
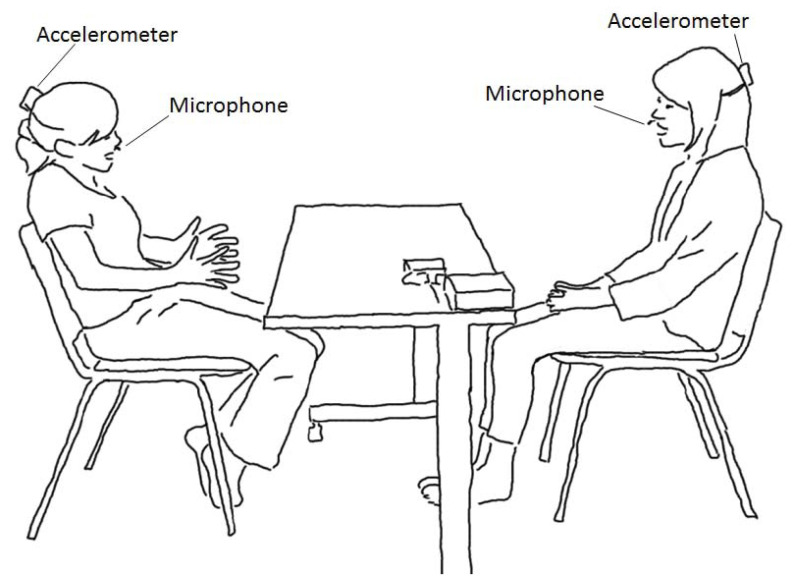
Illustration of setup for recording the dialogue. The client sits on the left and the therapist sits on the right with a table between them.

**Figure 2:**
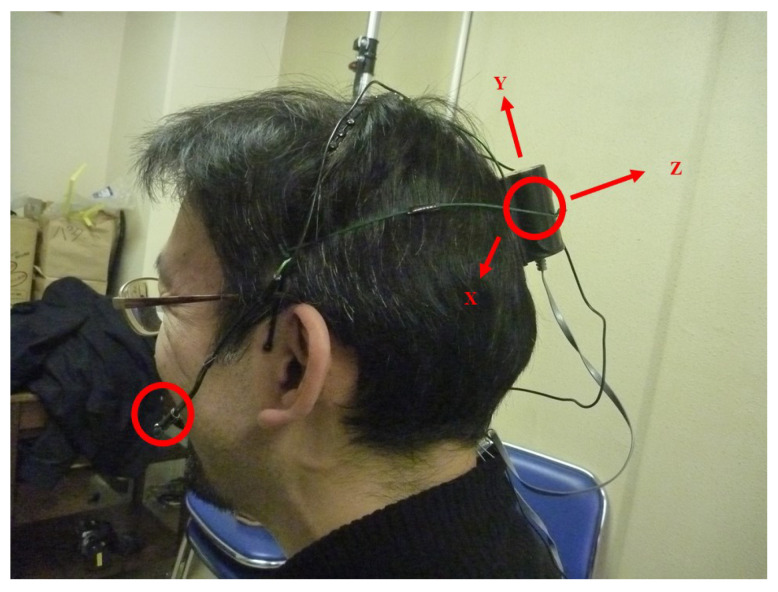
A microphone (DPA 4060-108BM) was placed near the mouth for high-quality recording. A triaxial analog accelerometer (Kionix KXM52-1050) in a protection case was mounted in the occipital region. The top face of the sensor was directed toward the Z-axis. The microphone and the sensor were directly wired to an 8-channel A/D recorder (NF circuit block, EZ7510) through coaxial and LAN cables.

All the interviews in the sessions were video-recorded. The participants could decide whether to use the video camera and sensor system described here. The participants faced each other; the video camera was placed on their side, and it captured images of their whole bodies as illustrated in Figure 1. In this study, speech data were recorded using a high-quality microphone (DPA 4060-BM) and head-movement data were acquired by triaxial head-mounted accelerometers (Kionix KXM52-1050) as shown in Figure 2. The analog signals were obtained through 4 channels per participant, 8 channels in total. The microphone and sensor were directly connected with wire cables to an 8-channel data recorder (NF circuit block, EZ7510) which enables A/D conversion from 0 Hz (DC) and can measure head movements with very low frequency components. Synchronization is important to consider multiple sensors [26]. The aforementioned setting allowed perfect synchronization between all speech sounds and acceleration data from both participants. The sampling rates were 50-kHz in the first session and 20-kHz in the second session (Although the upper limit of the frequencies differs, both sampling frequencies provide sufficient bandwidth, and this difference does not affect the analysis).

In the following analysis, we used only the acceleration along the vertical axis (Y-axis as shown in Figure 2) that can detect nodding by the mounted sensor. The sensors, which were 1 square centimeter in size, were placed on plates and covered by slightly larger protection cases. The cases were fixed to the heads using metal headbands. They were not seen by the other interlocutors as they were mounted in the occipital region. Due to the non-invasive characteristics of our sensor mounting systems, the participants reported that they could maintain a natural dialogue. Further, we did not observe any qualitative difference between dialogues with and without the sensor system. The recorded data had been reported in [27]. The participants moved their heads vertically before recording to check if vertical movements were recorded through the Y-axis channel as shown in Figure 2 and that the measured values did not go off the scale. Although the reference 1 gravitational acceleration was recorded using the recorder before the session, the information was not used for calibration because our analysis rely on the relative acceleration values and absolute values were not needed.

### 2.2. Nodding Counts

First, we manually coded the occurrence of the nods in two counseling cases. That is, the head nods were identified by a human annotator. Head nod counts were compared during different stages of the dialogue. Here, a qualified psychotherapist, who did not participate in the dialogue, used ELAN annotation software (https://archive.mpi.nl/tla/elan, accessed on 28 April 2021) [28] to annotate the nods for both recordings manually. Each nod was identified as a segment from the beginning to the end of the head movement. The semantic action of nodding comprises either a reciprocating motion or a series of swinging motions. That is, in coding, we treated continuous up-and-down head motions as nods. We did not account for differences in amplitude of head movements when counting head nods. The counts were plotted as a histogram with a bin width of 50s; then, the bin size was reduced. There are different types of head nods [29,30,31]; however, in our analysis, we considered all head nods without distinction because categorization of nods makes it difficult to identify trends with each category being quite sparse.

### 2.3. Head Movement Synchronization Degree

Here, we deal with the degree of the synchronization of head movements for the participants based on the accelerometer sensor’s time-series data, as described in Section 2.1. This was to determine whether therapists coordinate their head movements with the clients’ and if the degree of synchronization is associated with any events in the counseling sessions. It has been reported that head movements are associated with the long-term outcome of psychotherapy whereas body movements affect the short-term outcome [32]. Our case studies are on individual interview sessions, which are considered as short-term activities. However, we were interested in the process of psychotherapeutic interviews rather than the outcomes and we believe that head movements can offer useful information on the dialogue process. The therapists sometimes moved their heads simultaneously with those of the clients, whereas in other situations, the movements were delayed. The difference may be due to the different tasks being performed during the session. The acceleration signals of the head in the upward and downward directions were used as they were, regardless of whether they were head nods or not. Although synchronization has been studied in psychotherapy domain, many studies measure entire bodies or parts of bodies by image processing [33,34,35,36]. By using an accelerometer, we can consider the synchrony of specific parts of the body. Acceleration data were used as measures of head movements. The accelerometer outputs analog waveforms corresponding to the acceleration values along three orthogonal axes (X, Y, and Z). For the measurement setup, the head movements of interest approximately corresponded to the Y-axis values.

Cross correlation was calculated between the Y-axis data derived from the two accelerometers, one on the therapist and the other on the client. The raw data were resampled at 1 kHz; they were then filtered using a 60 Hz notch filter and a lowpass filter to reduce hum and high-frequency noise. Denoting the denoised signal of length *N* as $s=(s_{1},s_{2},...,s_{i},...,s_{N})$, that is, the Y-axis value of the three-dimensional vector of three axes, the normalized signal is as follows:

(1) $$x=\frac{s-\bar{s}}{||s-\bar{s}\left|\right|}$$

where

(2) $$\bar{s}=\frac{1}{N}\sum_{i=1}^{N}s_{i}\;\left(\mathrm{average}\right)$$

and

(3) $$||s-\bar{s}\left|\right|=\sqrt{\frac{1}{N}\sum_{i=1}^{N}{\left(s_{i}-\bar{s}\right)}^{2}}\;(\mathrm{standard}\;\mathrm{deviation}).$$

A window function w of size 500 ms was applied to the normalized signal x to calculate the maximum absolute values of cross correlation and delay for each frame (We did not find a notable difference when we changed the window size to 100 or 1000 ms). The window function was shifted from the start to the end of dialogue every 100 ms; thus, we obtained the cross-correlation time series.

The segment extracted by windowing is denoted as $x_{w}=x\cdot w$. At time frame $t_{f}$, time series $x_{w_{1}}\left(t_{f}\right)$ and $x_{w_{2}}\left(t_{f}\right)$ were obtained for the client and therapist, respectively. At $t_{f}$, with *n* as the data index number for every 1 ms, cross correlation is calculated as follows:

(4) $$R_{w}\left(t_{f},n_{\tau }\right)=\sum_{n=-\infty }^{\infty }x_{w_{1}}\left(t_{f},n+n_{\tau }\right)\cdot x_{w_{2}}\left(t_{f},n\right)$$

where $n_{\tau }$ is the lag between the acceleration of the client’s and therapist’s head. The maximum absolute cross correlation value is given as follows:

(5) $$r\left(t_{f}\right)=\max_{n_{\tau }}\left(\right|R_{w}\left(t_{f},n_{\tau }\right)\left|\right).$$

The lag that corresponds to this maximum value was obtained but not used in the analysis (When two time series after windowing are the same at $n_{\tau }$, cross correlation has a maximum value at $n_{\tau }=0$ ms. In our case, the maximum values were observed at approximately $n_{\tau }=\pm 25$ ms). To measure the relative degree of synchrony, the cross-correlation values of all frames between the first and last three minutes of the session were normalized by the maximum value. The first and last three minutes were excluded because these segments contained greetings and bowings that usually lead to strong synchrony; those cross-correlation values are too large to be used for normalization.

## 3. Results

### 3.1. Analysis of Nodding Counts

**Figure 3:**
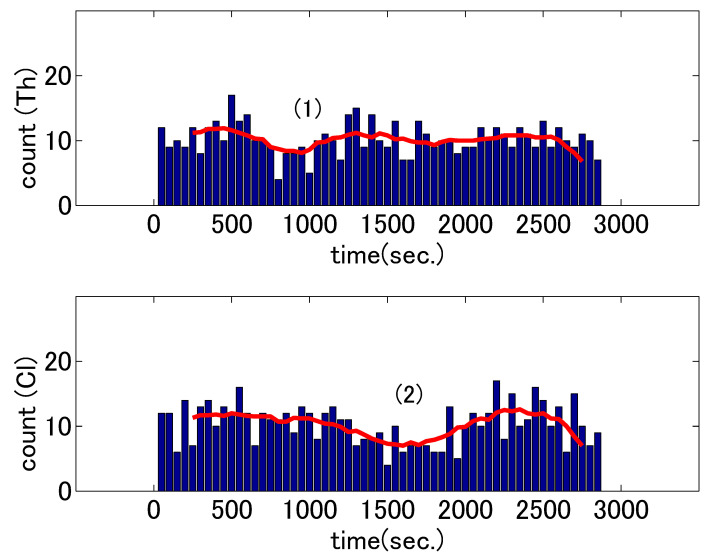
Nodding counts for the therapist (Th) and client (Cl) during the first dialogue. The bars represent the counts and the line corresponds to the eleven-point average values. The numbers in parentheses correspond to the numbers in Table 2.

**Figure 4:**
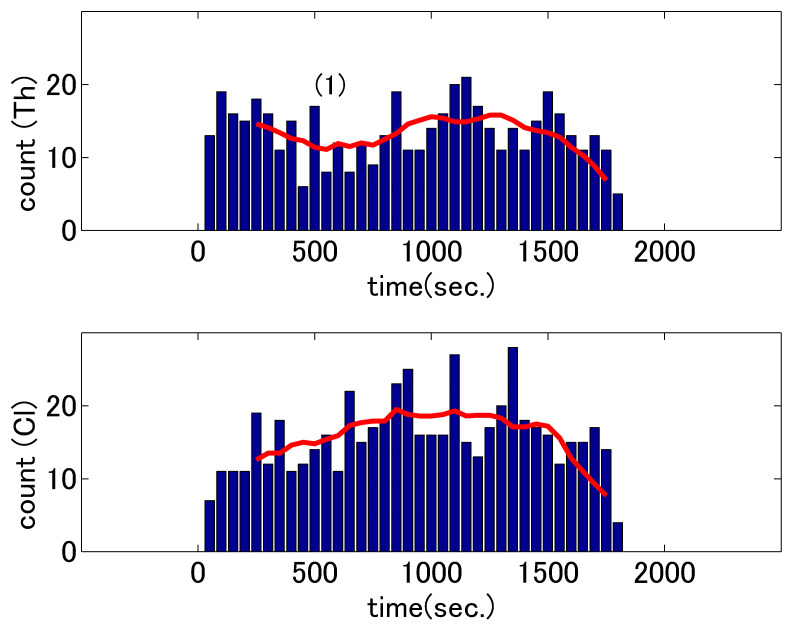
Nodding counts for the therapist (Th) and client (Cl) during the second dialogue. The bars represent the counts and the line corresponds to the eleven-point average values. The number in parentheses correspond to the numbers in Table 3.

We performed two analyses–head nodding counts and sensor signal correlation. We present the results on the first analysis using manually annotated head nod labels here to observe any patterns in the two psychotherapy cases. The changes in nodding counts of the two dialogues (based on the coding) are shown in Figure 3 and Figure 4. The upper and lower graphs in each figure correspond to those for the therapists and clients, respectively. The height of each bar represents the counts in the 50 s segments and the horizontal axes represent the time in seconds from the beginning of the session. To understand the overall trends rather than individual values in the time segments, we smoothed the counts by averaging the five preceding and five subsequent bins around the target bin and plotted these averages as lines over the bars.

The first analysis revealed that there were M-shaped changes in the therapist’s nodding counts in the first counseling dialogue, as shown in the upper graph in Figure 3. From the start of the dialogue, the counts increased and then subsequently dropped. The counts increased again before decreasing toward the end of the session. The initial increase and final decrease can be considered as natural changes that occur at the start and end of psychotherapeutic dialogues. In the beginning, the relationship between therapists and clients is not established; the interaction tends to be inactive. At the end of the dialogue, there are few topics to discuss and therefore there is less interaction. However, the reason for the drop in the middle of the dialogue is not clear. It is also interesting that the drops timings differed for the therapist and client. Accordingly, we closely investigated the events during these video segments. Description (1) in Table 2 shows the dialogue content when the therapist’s nodding counts were the lowest in the first dialogue. The therapist’s lowest nodding count occurred when the client uttered “I’m not characterized as a parent,” referring to the results of a personality test he had taken. The drop might have occurred because the client humbled himself by talking about their immaturity. The therapist might have wanted to urge them to reconsider and could not respond with an affirmative nod. The dialogue content for lowest client nodding count are shown in (2) in Table 2. The first time the client’s lowest count occurred when the therapist complimented the client by saying, “You can see yourself very well”; the client responded humbly by saying, “Ah, O, only this time.” The drop seemingly occurred when the therapist was working on the client, but the client could not accept her advice. One basic function of head nodding is to show the listeners’ agreement with speakers; however, the therapist could not use the nods in such a way. According to research on verbal interaction, expressions of disagreement are preferred over ones of agreement in evaluative utterances, such as humble or complimentary ones in contrast to ordinary utterances [37]. A similar phenomenon occurred non-verbally in the first dialogue; in response to the therapist’s compliment in (2); the client suppressed a head nod of functional agreement and responded with a hand gesture of disagreement by waving their hand in front of him. These two counts drops occurred in different sequences; however, both happened when the counseling entered a deeper stage of dealing with the client’s problem. In that stage, utterances to which the listeners would not or could not agree occurred. This implies that the nodding pattern changed when the counseling shifted from the information gathering stage (which involves simple questions and answers) to the stage in which introspection played a central role; the latter stage involved the client’s monologue-like utterances.

The upper graph in Figure 4 shows that the nod count of the expert therapist also changed in an M-shaped fashion. The dialogue content at the time of the second therapist’s lowest nodding count is shown in (1) in Table 3. Until then, the client had spontaneously provided details of the problem and tried to determine a solution; however, she failed to find a solution in her mind. From that point, the therapist led the session. This shift in dialogue stages—from the therapist listening to the client to asking questions to the client—might be reflected in the therapist’s pattern of nodding counts. Such shift of communicative activities between listening and asking question is often observed in solution-focused therapies that therapists practice in current cases [38].

For the client interviewed by the expert therapist, an inverted U-shaped nodding count pattern was observed (the lower of the two graphs in Figure 4). The second client’s highest nodding count occurred when the therapist was about to take the initiative during the counseling session. There was no drop in count on the client’s side, because the client did not engage in introspection but continued interacting with the therapist as their dialogue deepened. From the viewpoint of expertise, the first dialogue was led by the client and the inexperienced therapist simply reacted. By contrast, the second dialogue was coordinated by the experienced therapist, who was focused on problem solving rather than letting the client analyze the problem herself.

### 3.2. Analysis of Head Movement Synchronization Degree

**Figure 5:**
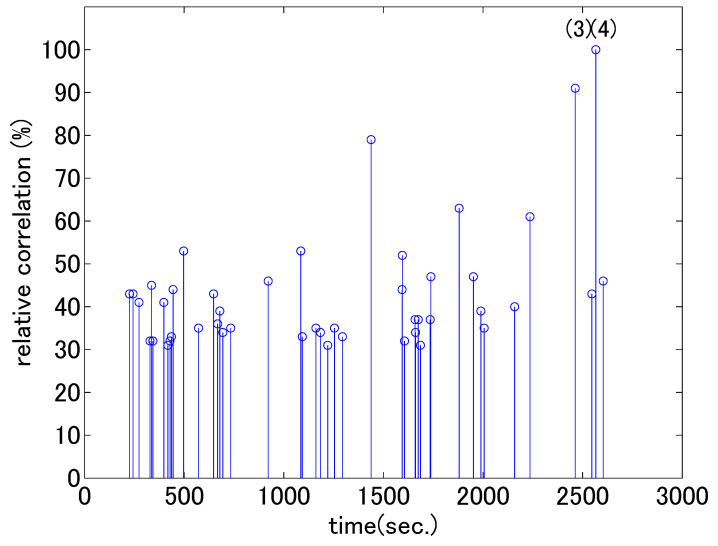
Cross-correlation values of head acceleration in the first dialogue (values greater than 30% of the maximum are shown). The horizontal axis corresponds to time frame $t_{f}$ in Equation (5). The numbers in parentheses correspond to the numbers in Table 2.

**Figure 6:**
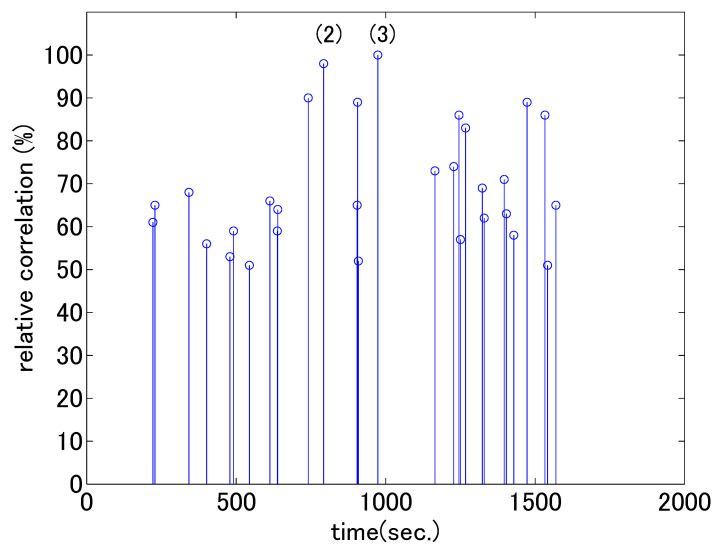
Cross-correlation values of head accelerations in the second dialogue (values greater than 50% of the maximum are shown). The horizontal axis corresponds to time frame $t_{f}$ in Equation (5). The numbers in parentheses correspond to the numbers in Table 3.

In this section, we present the results of the second analysis using accelerometers to observe any notable moments in the two psychotherapy cases. As explained in Section 2.3, we normalized the time series for individuals and used the degree of synchronization between the vertical head movements of the two people. Figure 5 and Figure 6 show the synchrony of head movements between the therapist and client. The horizontal axes represent the time from the beginning of the session. The height of each bar represents the maximum cross correlation value $r(t_{f})$ between the therapist’s and client’s greatest acceleration values in each analysis window; these are calculated by Equations (4) and (5) in Section 2.3. For ease of visual interpretation, we set the threshold values as 30% and 50% of the maximum value in each figure and displayed bars higher than the thresholds. In the previous analysis in Section 3.1, a gradual change in nodding counts was observed according to shift in dialogue stage. In this analysis, we see strong head movement correlation at certain moments.

In the first dialogue, the highest synchrony occurred at the end of the dialogue, when the client mentioned how nice their parents were, as described in Table 2 (3) and (4). The therapist was trying to enhance the client’s positive perception through strongly correlated nodding. For each of the client’s utterances, the therapist reacted with clearly identifiable nodding movements. The increase in synchrony when the positive attitude of the client is observed coincides with the reported patterns where rhythmicity occurs when the dialogue flows positively [39]. In the second dialogue, high synchronies occurred at two instances in the middle of the dialogue. The contents of the interactions are shown in (2) and (3) in Table 3. In interaction (2), the client who had previously been replying negatively eventually replied positively. At that point, the therapist seized the chance and tried to reach an agreement by nodding her head. In interaction (3), the therapist checked the facts surrounding the problem and asked for confirmation of each utterance of the client. The confirmation was accompanied by nodding. After this interaction, the client started talking in a lively manner.

Through content analysis, we identified interesting points at which head movements were synchronized differently for the two therapists. In the counseling by the novice therapist, head movements became synchronized as the client started to talk freely, whereas in the counseling by the experienced therapist, head movements became strongly synchronized when the therapist started to intervene in the client’s problems.

## 4. Discussion

### 4.1. Automatic Annotation of Head Nods

The automatic classification of head movements has been explored. Head movements are not the same as head nods. There are multiple functionalities for head movements. For example, Otsuka defined 32 function categories including 13 related to speaking, 12 related to reacting, and seven related to other functions [40]. They argued that there is no fixed one-to-one relation between kinetic features and functions. This property of head movements may make the automatic annotation of head nods difficult. For example, 0.68 F1 score for head movement detection, and 0.40 for head movement classification into four classes have been reported using visual, acoustic and textual features [41]. The result that the recall value for automatically detecting nods is 20.81% suggests that there is much scope for improvement. Although manual annotation is time-consuming, it offers some benefit to practitioners. It is observed that the participating therapists learn from annotating counseling sessions. In psychotherapy, micro analysis focuses on the occurrence of activities within a small time interval. For example, the verbal content of two different psychotherapeutic schools were compared in [38]. The process of annotating the counseling video can be regarded as a simplified form of micro analysis.

### 4.2. Channels of Case Reflection

**Figure 7:**
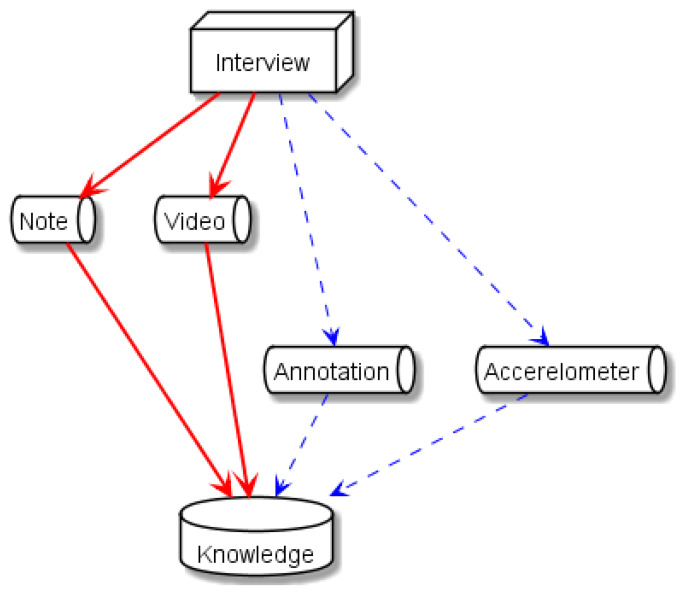
Reflection process through different analysis channels.

Psychotherapeutic interviews are diverse as the problems of each client are unique, the personalities of clients are different, the skill and style of therapists varies, and the environments of the counseling vary such as schools, local society, and workplaces. Therefore, even though the formal training on generic knowledge is given to the psychotherapist, such as the three-step structure of the interview process, which includes exploration, insight, and action [42], therapists must work on their own cases in the form of reflection for skill development. Reflections involve reviewing the previous counseling sessions to obtain insights on the interview process. The different forms of reflection include self-reflection (SR), group reflection or feedback, and supervision. SR is considered as a powerful tool to improve and develop the skills of therapists [43]. It can be done by examining the notes taken by the therapist during the interview sessions or by viewing the videos of the interview sessions. Video feedback is an effective method for training in which therapists can objectively examine the behaviors of both the client and the therapist [44]. Video reflection can be assisted by technologies and attempted in similar domains, such as coaching [45]. These commonly used channels are indicated by the two arrows in Figure 7. The channels we introduced in this study are indicated by the two right arrows in Figure 7. They are externally provided to practitioners by analysts. In our study, the annotation was assigned to head nods, and accelerometers were used to obtain head movement data. The annotation channel is not limited to head nods. Manual annotations for other modalities, such as verbal content [46] and hand gestures [47], can also be utilized for reviewing. Similarly, the accelerometer channel can be enhanced with other sensors. For example, blink detection sensors were combined with accelerometers [48]. The results of automated speech analysis based on the video recordings can be added to our head-nodding behavior analysis. It has been reported that the audio information during counseling sessions recorded using a far-field microphone can be utilized to predict counseling outcome [49].

### 4.3. Therapist Feedback on Results

Various forms of therapist training have been attempted [50]. However, lack of effective training is considered as a major barrier to deliver counseling [51]. We examined the utility of group reflection with our micro analysis on head movements for deepening the insights on counseling practice. We conducted a workshop with a self-learning group of therapists, including the therapists who participated in our study, and collected their opinions on the results of our analysis. Therapists typically organize reflective sessions after their psychotherapeutic interviews as part of their learning activities. During such self-organized sessions, they often discuss dialogue contents, such as what the participating therapist should have said or the interpretations of the client’s problem description. By contrast, during the workshop, we focused more on the way in which the therapists said what they said and the way they pursued their counseling by indicating the highlighted interactions described in the previous sections. The participating therapists mentioned that they did not regard and were not observant of head movements while they conducted counseling; however, they did understand that dialogue structures in terms of head movements existed, as revealed by the analysis. As for the second dialogue, we found a gradual shift from the information-collection to problem-solving stages, as discussed in Section 3.1. The therapist confirmed that she was going to take the initiative during period (1); this was verified by the nodding count analysis. A non-participating therapist noted that two similar questions were asked during a single counseling session. The questions where related to the goal of the counseling, i.e., what the client wanted to achieve. In general, such a question is asked only once. The first question was at 415 s and the second was at 606 s. The nodding counts increased after the first question and increased even further after the second question. This observation stimulated the therapist to interpret the different roles of two verbally similar questions: the first was intended to initiate the information collection process from the client, and the second question was intended to test the idea for a solution. These are examples of possible applications of our analysis framework.

Ideally, the observations obtained by our analysis may lead to better counseling practices. However, as psychotherapy is a complex endeavor, an immediate measurable outcome cannot be expected. One clear achievement is that the participating therapists have gained insight into the reflection on individual cases with additional channels, which go beyond verbal manifestation in their counseling.

## 5. Conclusions

To obtain insights for better interactions in the mental health domain, we analyzed head nodding and movement patterns in task-oriented psychotherapeutic dialogues for better reflection on specific interview sessions. By annotating nods using videos of client–therapist interactions, we found that patterns of nodding count changes through the course of counseling. The degree of synchronization of head movements between the two participants was measured using accelerometers. We found that distinctive communication events occurred when head movements were strongly correlated. In addition, by comparing therapists with different degrees of expertise, we identified the possibility that head nodding patterns vary according to the skill level of the therapist. These insights were not obtained when the dialogue was watched for the first time and the focus was on the verbal content. By considering nodding behavior based on temporal variations, notable segments of the dialogues emerged. When the amount of data analyzed is increased, it becomes possible to identify which head nod patterns or head movement patterns can be generalized and which patterns are unique to specific cases. If we extend our observations by collecting more annotations from different annotators on each case, we can measure the variability of head nod annotation. Interpretive targets such as humor [52] involves large varieties of annotators. Although head nod is basically a physical target, there are still semantic interpretation involved and some variations in annotation results are expected between annotators. When such varied annotations are used but cannot extract moments that were identified by our second analysis using accelerometer data, the utility of objective sensor-based method to complement human judgments will become clearer. By applying some agreement metric to the varied human annotations, we can examine if we can obtain stable patterns using multiple annotations either in linguistic events [53] or in time series [54]. When mapping sensor signals to semantic categories, the variability of sensed data should be examined [55]. The repeated measurements of dialogues in the experimental setting can help identify the variations in the stability of data from sensor-based methods.

Our sensor systems can be enhanced by adding more sensors for more information and change the connection from wired to wireless for more flexibility. In such a method, a multi-unit synchronized system may be utilized [56]. The IMUs can be simulated by processing video data to some extent [57]. Our analyses can be enhanced by incorporating head movement pattern mining [58] in addition to the current signal-based approach.

## Figures and Tables

**Table 1:** Overview of dialogue datasets.

	Time	Therapist (Experience)	Client
Dialogue 1	2850 s	Female (novice: 1 year)	Male
Dialogue 2	1789 s	Female (experienced: 11 years)	Female

**Table 2:** Contents of interactions in the first dialogue highlighted by the analysis (Therapist: Th, Client: Cl).

	Sec.	Content
(1)	950	Cl: Well, I’m not characterized as a parent.
(2)	1600	Th: Great! You can see yourself very well.
		Cl: Ah, O, only this time.
(3)	2463	Cl: Such, (pause) well, my parents brought me up,
		and I feel kind of, well, happy about it.
(4)	2566	Cl: However, my parents were, well, different.
		They, well, stood up for their children earnestly.

**Table 3:** Contents of interactions in the second dialogue highlighted by the analysis (Therapist: Th, Client: Cl).

	Sec.	Content
(1)	550	Cl: I’m wondering what I should do. (folding her arms)
		Th: So, she does not take it out on someone outside.
		(scratching her head)
(2)	793	Cl: They left me out; something like [Cl] is okay already.
		(laughing)
		Th: (nodding deeply) So, they do not worry about you.
		Cl: That’s right.
(3)	974	Th: It’s something like evacuation.
		Cl: Yes, evacuation.
		Th: You can do that.

## Data Availability

The data are not publicly available due to the nature of the dialogues.
